# Multiparametric mapping of white matter reorganizations in patients with frontal glioma‐related epilepsy

**DOI:** 10.1111/cns.14322

**Published:** 2023-06-28

**Authors:** Simin Zhang, Fei Zhao, Xibiao Yang, Qiaoyue Tan, Shuang Li, Hanbin Shao, Xiaorui Su, Qiyong Gong, Qiang Yue

**Affiliations:** ^1^ Department of Radiology, Huaxi MR Research Center (HMRRC) West China Hospital of Sichuan University Chengdu China; ^2^ Department of Radiology West China Hospital of Sichuan University Chengdu China; ^3^ Research Unit of Psychoradiology Chinese Academy of Medical Sciences Chengdu China; ^4^ Functional and Molecular Imaging Key Laboratory of Sichuan Province West China Hospital of Sichuan University Chengdu China; ^5^ Huaxi Glioma Center West China Hospital of Sichuan University Chengdu China; ^6^ Division of Radiation Physics, State Key Laboratory of Biotherapy and Cancer Center West China Hospital of Sichuan University Chengdu China; ^7^ Department of Radiology West China Xiamen Hospital of Sichuan University Xiamen Fujian China

**Keywords:** glioma related epilepsy, graph theory analysis, structural network, topological metrics, tractometry, white matter

## Abstract

**Aims:**

Epilepsy is a common symptom in diffuse lower‐grade glioma (DLGG). The specific role of white matter (WM) alteration in patients with glioma‐related epilepsy (GRE) is largely unknown. This study aims to investigate the reorganization of WM tracts and changes in structural networks related to GRE.

**Methods:**

Diffusion‐weighted images were collected from 70 patients with left frontal DLGG (GRE = 33, non‐GRE = 37) and 41 healthy controls (HC). Tractometry with TractSeg was applied to segment tracts and quantify fractional anisotropy (FA) along each tract. Structural network was constructed using constrained spherical deconvolution and probabilistic tractography. FA and network properties were compared among three groups.

**Results:**

Compared with HC, both GRE and non‐GRE showed decreased FA in contralateral inferior fronto‐occipital fasciculus, superior longitudinal fasciculus II and arcuate fasciculus, increased nodal efficiency in contralateral nodes of frontal–parietal and limbic networks, whereas decreased degree centrality and betweenness centrality in nodes of dorsal temporal lobe and rostral middle frontal gyrus (rMFG). Additionally, when compared GRE with non‐GRE, increased FA in contralateral corticospinal tract (CST) and lower betweenness centrality in paracentral lobule (PCL) in GRE (all *p* < 0.05 after Bonferroni correction).

**Conclusion:**

This study indicates that patients with left frontal DLGG exhibit complex WM reorganization, and the altered regions mainly concentrated in the language, frontal–parietal and limbic networks. Moreover, the preserved integrity in contralateral CST and server decreased nodal betweenness in PCL may be potential neuroimaging markers underlying the occurrence of presurgical seizures of GRE.

## Graphical abstract

This diffusion study applied comprehensive set of sophisticated white matter microstructural parameters to investigate WM alternations in patients with glioma‐related epilepsy, which may provide new insights into the pathophysiological mechanisms underlying this disease.

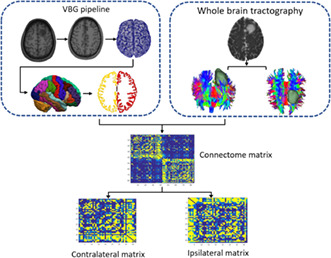

## 1 INTRODUCTION

Glioma‐related epilepsy (GRE) is characterized by symptomatic epileptic seizures secondary to gliomas,^1^ particularly in patients with diffuse lower‐grade glioma (DLGG) invading the frontal lobes.^2^, ^3^ Due to the instability, unpredictability and close association with glioma progression/recurrence,^4^ GRE profoundly impact patients' prognosis and quality of life.^1^ However, the pathogenesis of GRE is multifactorial and still not fully understood, which poses a significant challenge for antiepileptic therapy in patients with newly diagnosed glioma.^5^

Recent evidence emphasizes the role of white matter (WM) in the pathogenesis of epilepsy.^6^ It has been demonstrated that pathological changes including WM myelination, axonal integrity, and cellular composition were observed in ex vivo and post‐mortem studies of epileptic patients.^7^ Diffusion tensor imaging (DTI) is increasingly used for noninvasive and quantitative assessment of WM abnormalities, which provide us a valuable choice for investigating the pathophysiological mechanisms underlying GRE.

Epileptic seizures could trigger the alternations of WM fiber Tracts.^8^, ^9^ Study confirmed WM microstructural reorganization in non‐lesion epilepsy.^10^ As for DLGG, it is a slow‐growing neoplasm that infiltrates alongside the WM fibers, which may result in impaired or reorganized WM structure beyond or even distal to the tumor border,^11^ and thus may affect the patient's postoperative recovery. Nonetheless, when DLGG combined with epileptic seizures, how did the WM reorganize was still poorly understand. A prior study, utilized deterministic tractography algorithm, revealed that DLGG patients with epilepsy are more likely to show structural and connectivity aberrations in various distant brain regions, indicating the potential correlation between epileptogenesis and microstructural changes in DLGG patients.^12^ A recent Tract‐Based Spatial Statistics (TBSS) study investigated the effects of GRE on WM and revealed that the changes were mainly concentrated in the ipsilateral WM tracts.^13^ TBSS incorporates a skeletonization step that mitigates residual misalignment and eliminates the need for data smoothing^14^; however, this technique is still has difficulties in precisely define the actual tract location of fibers in an individual.^15^ On the other hand, epilepsy is thought as a neurological network disorder,^16^ most epileptic seizures are related to impair functional or structural connections in several networks.^17^, ^18^ Graph theory‐based approach provides a novel framework to characterize the topological organization of the brain's structural and functional networks. In this approach, the brain can be modeled as a complex network represented graphically by a collection of nodes (e.g., brain regions) and edges (e.g., connectivity).^19^ The topological organization of brain networks can be quantitatively described by a wide variety of measures.^20^ In particular, the nodal efficiency, degree centrality and betweenness centrality are important regional nodal metrics that quantify how important a node is within a network, and have been widely used to detect regional abnormalities of brain networks. However, the association between GRE and alterations in structural network and their topological attributes remains unknown.

Moreover, compared to the WM alternations in the ipsilateral hemisphere, based on the theory of neuroplasticity, the contralateral hemisphere may show reorganization and striking reconfigurations of WM architecture and network.^21^, ^22^ In this context, multiparametric mapping of WM alternations, especially subtle changes in the contralateral WM, is crucial for in‐depth investigation of the pathophysiological mechanisms of GRE.

Therefore, in the current study, we combined two level of diffusion MR analysis techniques to assess WM microstructural alterations underlying DLGG and GRE. Firstly, we employed TractSeg,^23^ which is a fully convolutional neural network method that able to accurately segment fiber tracts into bundles at individual level, thereby avoiding possible registration errors during mapping individual brains to standard space. Then statistical analyses were performed by evaluating FA along each of the 15 contralateral hemisphere tracts previously implicated in frontal glioma or epilepsy.^10^, ^13^ Second, we combined tractography with graph theoretical analysis to investigate large‐scale network measures. We hypothesized that the WM architecture and graph network would show compensation in the contralateral hemisphere.

## 2 METHODS

### 2.1 Participants

This study was approved by the ethics committee of West China Hospital of Sichuan University. Written informed consent was obtained from all participants. The inclusion criteria were as follows: (a) pathologically diagnosed with DLGG (refer to 2021 WHO criteria, i.e., grade 2 diffuse glioma with isocitrate dehydrogenase–mutated); (b) patients were aged ≥ 18; (c) DLGG involved in unilateral left frontal lobe and invasion had not reached the central sulcus; (d) with no evidence of shift of the midline structures (septum pellucidum, corpus callosum, third ventricle due to lesion mass effect, based on preoperative three‐dimensional T1‐weighted images (3DT1WI), T2‐weighted images (T2WI), gadolinium enhanced [Multihance, Braccosine] three‐dimensional T1‐weighted images (Gd‐3DT1WI) and fluid‐attenuated inversion recovery (FLAIR); (e) without any history of treatment before undergoing MRI scan; (f) epilepsy symptoms were evaluated according to the 2017 International League Against Epilepsy (ILAE) guidelines. Finally, 70 patients with left frontal DLGG were included, among them, 33 patients had preoperative seizure onset while the other 37 had not, thereby divided into GRE group and non‐GRE group. Additionally, 41 healthy participants matched by age, sex, and education level were recruited as healthy controls (HC). The process of patients' enrollment was presented in Figure S1.

### 2.2 MRI acquisition

All imaging data were acquired using a 3.0 T system (Skyra, Siemens Healthineers) with a 20‐channel head coil. MRI sequences including: (a) 3DT1WI and Gd‐3DT1WI were obtained using magnetization‐prepared rapid gradient‐echo (MPRAGE): repeat time (TR)/echo time (TE) = 1630/2.29 milliseconds (ms), matrix = 256 × 256, flip angle = 8°; field of view (FOV) = 230 × 230 mm; slice thickness = 1 mm. (b) Diffusion‐weighted images (DWI) using a single shot, echo planar imaging sequence: TR/TE = 6000/93 ms, matrix = 192 × 192, flip angle = 90°, FOV = 229 × 229 mm, diffusion directions = 30, b = 0/1000 s/mm^2^, slice thickness = 3 mm. (c) T2WI: TR/TE = 4500/105 ms, matrix = 320 × 230, flip angle = 150°, FOV = 185 × 220 mm; slice thickness = 5 mm. (d) FLAIR: TR/TE = 6000/81 ms; matrix = 320 × 196, flip angle = 150°, FOV = 185 × 220 mm; slice thickness = 5 mm.

### 2.3 Glioma segmentation

We used a 3D U‐net architecture to automatically segment gliomas, which has demonstrated the reliability and efficiency in previous work.^24^ The detail segmentation processes are provided as Supplementary Information (Data S1). Finally, a mask encompassing whole tumor area was generated. The initial mask was visually assessed independently by two neuroradiologists (X.B.Y and H.J.Z, both with >10 years of experience in neuro oncology) using 3D slicer software (https://www.slicer.org/).

### 2.4 Image processing

Three dimensional T1 images were processed using virtual brain grafting (VBG) combined with Freesurfer (https://github.com/KUL‐Radneuron/KUL_VBG). VBG is a new workflow for reliable structural mapping of T1 images in the presence of glioma lesions, which enables realistic lesion filling and an accurate whole brain parcellation. As the first step, VBG creates the glioma lesion‐free T1‐weighted images using the glioma segmentation mask. Subsequently, brain parcellation utilizing Freesurfer's recon‐all command^25^ on the output lesion‐free image.^26^ Finally, anatomical segmentations including surface reconstructions and cortical parcellations are generated, following the Desikan‐Killiany (DK) atlas,^27^ resulting in 84 nodes.

DWI data were preprocessed using MRtrix3 (http://mrtrix.org), steps included denoising, correction of Gibbs ringing artifacts, head motion, eddy currents and bias field.^28^, ^29^

### 2.5 Data analysis

#### 2.5.1 Tractometry

White matter bundle segmentation was carried out in template space using TractSeg.^30^ Subsequently, tractometry analysis was conducted on the delineated tracts to assess the FA along 100 points of each tract (more details see supplementary method). Fifteen tracts of interest were identified including inferior fronto‐occipital fasciculus (IFOF), arcuate fasciculus (AF), anterior thalamic radiation (ATR), superior longitudinal fasciculus (SLF)‐I, SLF‐II, SLF‐III, corticospinal tract (CST), inferior longitudinal fasciculus (ILF), frontopontine tracts (FPT), Fornix (FX), uncinated fasciculus (UF), corpus callosum (rostrum (CC 1), genu (CC 2), rostral body (CC 3) and anterior midbody (CC 4)) to investigate further potential WM changes of fiber pathways in patients with GRE and non‐GRE in contralateral hemisphere.

#### 2.5.2 Connectome construction

**FIGURE 1:**
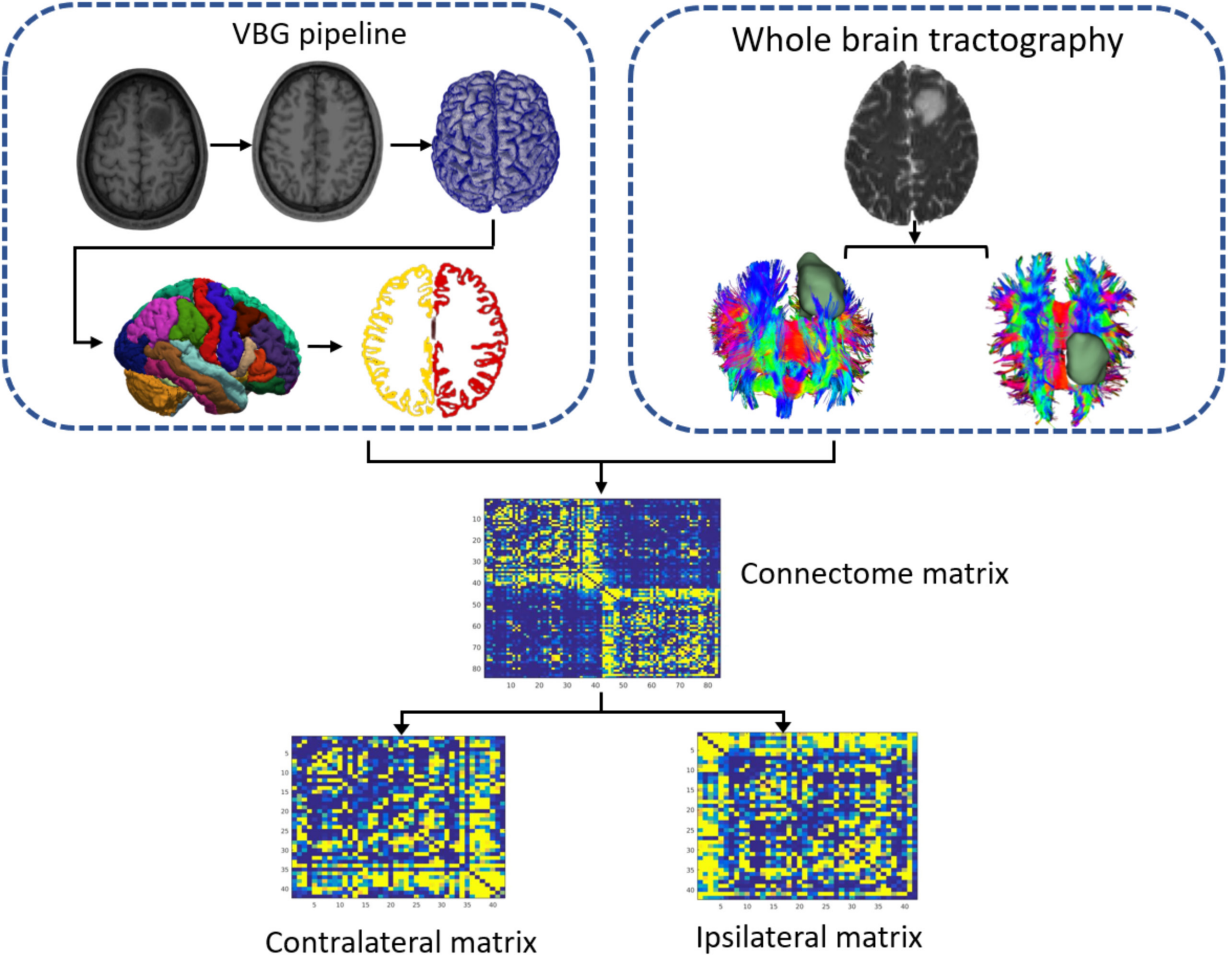
Diagram shows workflow for structural network construction. Whole‐brain parcellation and surface reconstructions in patient with DLGG were processed used VBG pipeline based on the 3DT1WI and segmented tumor mask. After whole brain tractogram generation using probabilistic tractography and SIFT filtering, an 84 × 84 adjacency matrix was generated. Further, the connectome matrices were divided into contra‐ and ipsilateral matrices. Abbreviations: DLGG, diffuse low grade glioma; SIFT, spherical‐deconvolution informed filtering of tractograms; VBG, virtual brain grafting.

Whole brain tractography was performed utilizing the multi‐tissue Constrained Spherical Deconvolution (CSD) tracking algorithm^31^ and SIFT filtering^32^ which were provided within the MRtrix3^33^ (more details see supplementary method). To define the nodes of the network, the DK atlas was used to divide the whole brain into 84 anatomical regions (42 for each hemisphere). The parcellation procedure was utilized the VBG combined with freesurfer which were mentioned in the subsection of image processing. Next, to define the edges of the network, “mean FA” of fibers linking any two nodes was calculated using the “tcksample” and “tck2connectome” commands in MRtrix3. Finally, a weighted and undirected 84 × 84 matrix for each participant was generated. After that, the whole‐brain connectome matrix was split into ipsilateral and contralateral matrices representing the two hemispheres separately using MATLAB (R2020b) Software, and a temporary 42 × 42 matrix was generated for left hemisphere (Figure 1). Then, the GRETNA 2.0.0 (https://www.nitrc.org/projects/gretna/) was used to calculate the nodal topological metrics including nodal efficiency, degree centrality and betweenness centrality of the WM networks. (Detailed definitions of the topological properties are shown in Table S1).

### 2.6 Statistical analyses

The Shapiro–Wilk test was used to assess the normality of all continuous variables (age, educational level, FA and nodal properties). The one‐way analysis of variance (ANOVA) or analysis of covariance analysis (ANCOVA) was used accordingly for normally distributed data, whilst the Kruskal–Wallis test was used for nonnormal distribution. In this study, all the continuous variables were distributed normally. Thus, an ANOVA test was used to compare age and education level among three groups. A chi‐squared test was used to compare sex ratio among three groups. Moreover, to compare astrocytoma ratio differences between GRE and none‐GRE group, chi‐squared test was used. A *p* value <0.05 was considered significant.

An analysis of covariance analysis (ANCOVA) was used to compare the FA across 100 points of each fiber tract and the AUC value of each nodal property located in contralateral hemispheres among the GRE, non‐GRE and HC groups. The Bonferroni was used to correct for multiple comparisons (*p* < 0.05, Bonferroni corrected). If ANCOVA showed significant differences, post‐hoc analysis was performed using Turkey test (FA metrics) or least‐significant difference method (nodal properties) to determine intergroup differences. The age, sex and educational level were set as covariates.

## 3 RESULTS

### 3.1 Clinical and demographic characteristics

Based on our inclusion criteria, in the GRE group, 33 patients were recruited (mean age, 40.58; age range, 18–66; sex ratio, 19 male/14 female), in the non‐GRE group, 37 patients were recruited (mean age, 44.54; age range, 20–71; sex ratio, 18 male/19 female) and in HC group, 41 subjects were recruited (mean age, 45.93; age range, 20–68; sex ratio, 23 male/18 female). No significant differences were found in age (*p* = 0.249), educational level (*p* = 0.500), or sex ratio (*p* = 0.718) among groups. There were no differences in 1p/19q codeletion ratio between GRE and non‐GRE (*p* = 0.522). Demographic and clinical characteristics are summarized in Table 1.

**TABLE 1:** General characteristics of the participants.

	GRE	Non‐ GRE	HC	*p* values
No.	33	37	41	NA
Age (mean ± SD), years	40.58 ± 15.10	44.54 ± 15.16	45.93 ± 12.8	0.249
Sex ratio, M/F, *n*	19/14	18/19	23/18	0.718
Education level, years	11.64 ± 3.05	11.08 ± 3.09	11.85 ± 2.72	0.500
Diagnosis				
Astrocytoma (IDHmut &1p/19q‐intact)	18	21	_	0.522
Oligodendroglioma (IDHmut &1p/19q‐codeleted)	15	16	_	
Seizure onset age, years	40.42 ± 15.02	_	_	_
Seizure duration prior surgery, days	57.85 ± 41.10	_	_	_
Seizure type, *n*				
Focal	17	_	_	_
Generalized	13	_	_	_
Unknow onset	3	_	_	_
Seizure frequency				
Low (only once)	26	_	_	_
Medium (2 ~ 3 times)	5	_	_	_
High (>3 times)	2	_	_	_

Abbreviations: GRE, glioma‐related epilepsy; HC, healthy control; IDHmut, isocitrate dehydrogenase mutation; NA, not applicable; non‐GRE = patients without glioma‐related epilepsy.

### 3.2 Categorical analyses

#### 3.2.1 Tractometry

**FIGURE 2:**
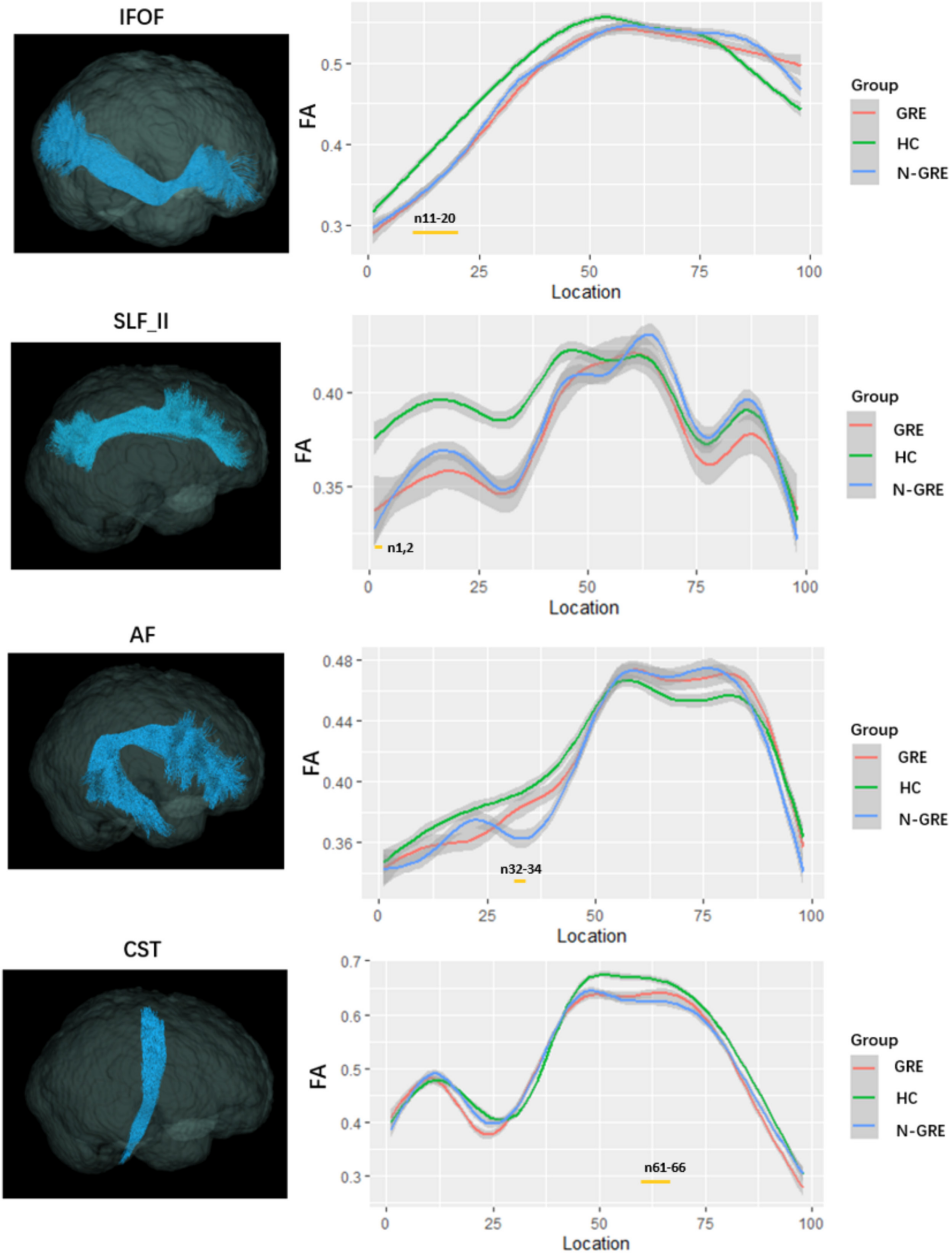
The plots of FA profiles of significantly altered fiber tracts among GRE, non‐GRE, and HC groups (Bonferroni correction, *p* < 0.05). The yellow bars under the profile indicate the regions of significant difference among three groups. Abbreviations: FA, fractional anisotropy; GRE, glioma related epilepsy; HC, healthy control; non‐GRE, patients without glioma‐related epilepsy.

The results of point‐wise level group difference were shown in Figure 2 and Table 2. Overall, the affected contralateral tracts included IFOF, CST, SLF‐II and AF. Significantly decreased FA were found in IFOF (node 11–20), AF (node 32‐34) and SLF‐II (node 1,2) in both GRE and non‐GRE patients compared with HC. Moreover, comparison of the two patient groups revealed decreased FA in nodes 61–66 of CST in non‐GRE group (All *p* < 0.05 after Bonferroni correction).

**TABLE 2:** The location of tracts showing significant group differences in FA among GRE, non‐GRE and HC.

Fiber track	Node	GRE	Non‐GRE	HC	ANCOVA		Post‐hoc test (*p* values)		
					*F* values	*p* values	GRE vs. HC	Non‐GRE vs. HC	GRE vs. non‐GRE
R. IFOF	11	0.335 ± 0.047	0.334 ± 0.053	0.372 ± 0.047	6.674	0.0019	0.008	0.004	0.995
	12	0.339 ± 0.045	0.341 ± 0.055	0.378 ± 0.049	6.260	0.00278	0.008	0.007	0.996
	13	0.343 ± 0.046	0.345 ± 0.056	0.384 ± 0.048	7.529	0.00092	0.003	0.003	0.986
	14	0.349 ± 0.057	0.347 ± 0.044	0.392 ± 0.047	9.026	0.00026	0.001	0.001	0.979
	15	0.351 ± 0.043	0.354 ± 0.058	0.399 ± 0.045	10.564	0.00007	0.0003	0.0004	0.977
	16	0.359 ± 0.058	0.356 ± 0.044	0.401 ± 0.044	11.407	0.00004	0.0002	0.0002	0.982
	17	0.363 ± 0.058	0.360 ± 0.041	0.412 ± 0.043	12.152	0.00002	0.0001	0.0001	0.978
	18	0.365 ± 0.041	0.366 ± 0.058	0.420 ± 0.043	13.195	0.00001	0.00008	0.0006	0.999
	19	0.370 ± 0.043	0.369 ± 0.059	0.423 ± 0.043	13.924	0.000005	0.00007	0.00002	0.998
	20	0.376 ± 0.045	0.374 ± 0.060	0.428 ± 0.045	12.969	0.00001	0.0002	0.00005	0.992
R.CST	61	0.626 ± 0.062	0.594 ± 0.171	0.671 ± 0.052	5.236	0.00695	0.247	0.00001	0.004
	62	0.650 ± 0.062	0.570 ± 0.062	0.664 ± 0.0506	5.814	0.00414	0.837	0.0005	0.007
	63	0.641 ± 0.049	0.576 ± 0.064	0.670 ± 0.0456	6.423	0.00241	0.469	0.0003	0.003
	64	0.638 ± 0.049	0.563 ± 0.065	0.669 ± 0.043	6.220	0.00288	0.372	0.00003	0.001
	65	0.629 ± 0.048	0.570 ± 0.063	0.656 ± 0.046	5.216	0.00707	0.873	0.001	0.006
	66	0.639 ± 0.057	0.563 ± 0.063	0.652 ± 0.048	4.667	0.01164	0.979	0.0002	0.002
R.SLF‐II	1	0.338 ± 0.076	0.332 ± 0.037	0.381 ± 0.051	8.593	0.00037	0.0001	0.001	0.908
	2	0.341 ± 0.077	0.332 ± 0.036	0.382 ± 0.048	8.935	0.00028	0.007	0.001	0.827
R.AF	32	0.363 ± 0.0426	0.360 ± 0.0426	0.398 ± 0.040	6.945	0.00152	0.004	0.001	0.880
	33	0.371 ± 0.0484	0.360 ± 0.044	0.400 ± 0.041	7.394	0.00103	0.007	0.001	0.719
	34	0.387 ± 0.055	0.373 ± 0.047	0.411 ± 0.044	7.325	0.00109	0.002	0.005	0.12

*Note*: The *F* and *p* values were obtained using ANCOVA, setting age, sex and education level as covariate.

Abbreviations: GRE, glioma‐related epilepsy; HC, healthy control; NA, not applicable; non‐GRE, patients without glioma‐related epilepsy; R.AF, right arcuate fasciculus; R.CST, right corticospinal tract; R. IFOF, right inferior fronto‐occipital fasciculus; R. SLF‐II, right superior longitudinal fasciculus‐II.

#### 3.2.2 Large‐scale network analysis

**FIGURE 3:**
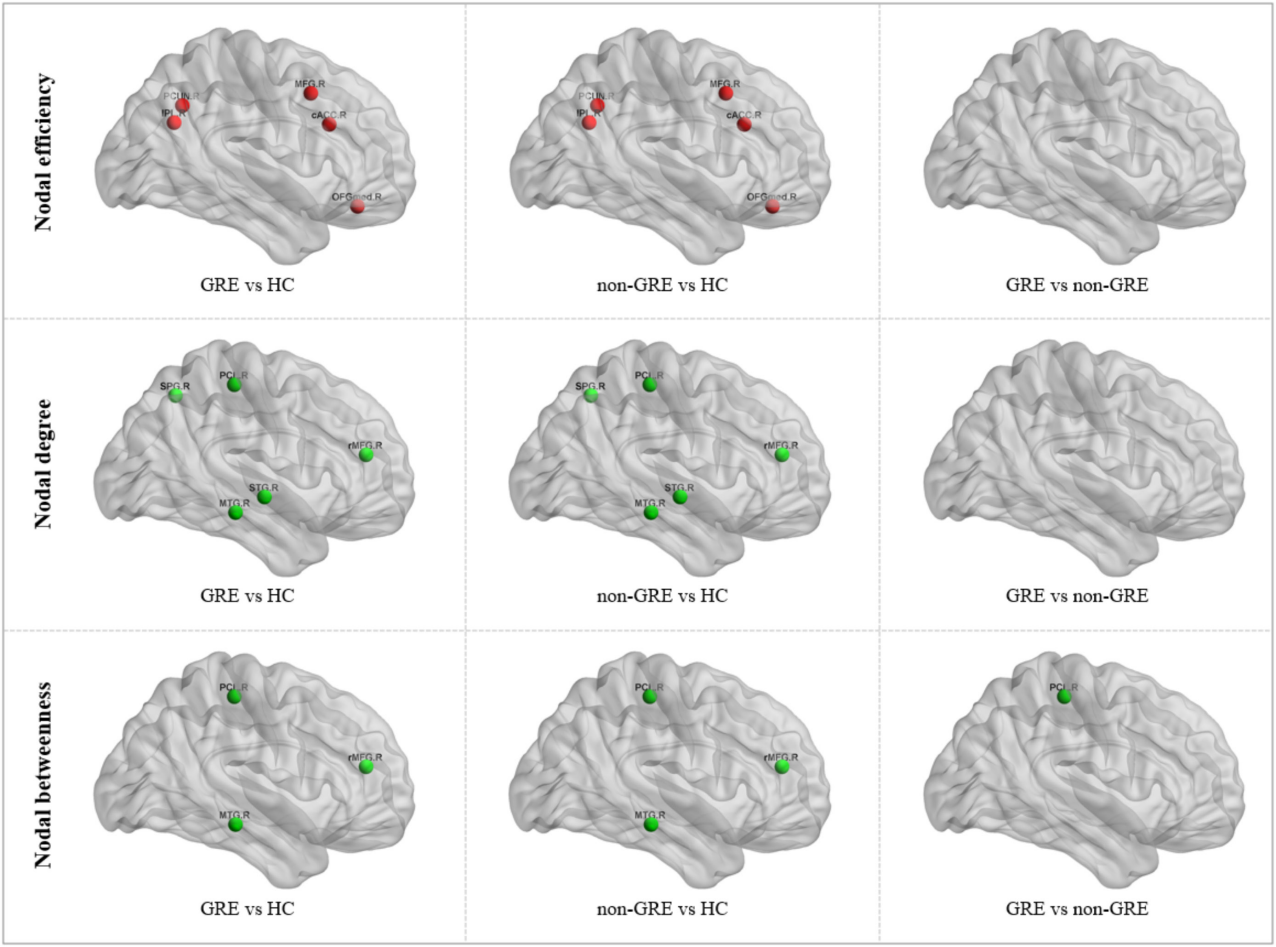
The differences in nodal properties of the structural networks across the GRE, non‐GRE, and HC groups. The nodes with significantly increased or decreased nodal efficiency, degree and betweenness are shown in red or green. Abbreviations: GRE, glioma related epilepsy; HC, healthy control; non‐GRE, patients without glioma‐related epilepsy.

Significant group differences (all *p* < 0.05 after bonferroni correction) in nodal topological properties are shown in Figure 3 and Tables S2‐S5. Regarding nodal efficiency, compared with the HC group, both GRE and non‐GRE groups showed higher node efficiency in the contralateral caudal anterior cingulate cortex (cACC), middle frontal gyrus (MFG), medial orbitofrontal cortex (mOFC), precuneus, inferior parietal lobule (IPL). However, there was no difference in nodal efficiency between the GRE and non‐GRE groups. As for degree centrality, compared with the HC group, both GRE and non‐GRE patients showed lower degree centrality in the contralateral middle temporal gyrus (MTG), paracentral lobule (PCL), superior temporal gyrus (STG), rostral middle frontal gyrus (rMFG) and superior parietal lobule (SPL). Comparison of the two patient groups revealed no significant difference. With respect to betweenness centrality, compared with the HC group, both GRE and non‐GRE patients showed lower betweenness centrality in contralateral MTG, rMFG and PCL. Moreover, GRE group exhibited a further significant lower betweenness centrality in PCL than non‐GRE group.

## 4 DISCUSSION

This diffusion MRI study investigated brain WM microstructure alternations in GRE and non‐GRE patients. For the WM integrity, we found significantly decreased FA in contralateral WM pathways related to language processing including IFOF, AF and SLF‐II in both patient groups. Furtherly, a more pronounced FA decrease in CST in non‐GRE compared to GRE. For the nodal properties, both GRE and non‐GRE patients showed higher nodal efficiency in contralateral nodes of the frontal–parietal and limbic networks, whereas lower degree centrality and betweenness centrality in nodes of dorsal temporal lobe and rMFG. Importantly, GRE group showed further reductions in betweenness centrality in the right PCL relative to non‐GRE, indicating that this node is susceptible to GRE. Therefore, our findings derived from multiparametric mapping of WM provide a new perspective for understanding the potential pathophysiology in GRE and non‐GRE patients.

### 4.1 Tractometry

Partly consistent with the current results, a prior DTI investigation utilizing TBSS revealed the majority of WM alterations in GRE localized in IFOF, SLF and superior corona radiata.^13^ However, TBSS is not able to accurately assign FA values to the same WM tract across subjects in a consistent way. To overcome above limitation, we employed Tractometry with TractSeg, a new method which can quantitively measure DTI metrics along with fiber tractography, and analyze microstructural alterations without the loss of orientation information.^30^ Notably, the current study focused on investigation WM reorganization pattern in in “normal‐appearing” area, thus, the contralateral hemisphere of each participant was analyzed.

An important finding in Tractometry analysis was both patient groups showed decreased FA in contralateral IFOF, SLF‐II, and AF, whereas these tracts showed no difference in patients' group (GRE vs. non‐GRE). The main attributing factor to the finding could be an influence of left frontal DLGG on the WM fibers. It has been established that IFOF, SLF‐II and AF played a crucial role in language function.^34^, ^35^ Particularly, according to the ‘dual stream’ theory of language processing, the IFOF, ILF and UF integrate information within the ventral stream where semantic and syntactic analyses predominate, whereas the SLF and AF belong to the dorsal stream where phonological and articulatory processing occur.^36^ Therefore, damage to any portion of the above‐mentioned tracts may produce conduction aphasia, which is characterized by fluency and paraphasia in spontaneous speech, with markedly impaired repetition of words and phrases, and usually shows difficulty in naming.^37^ Indeed, previous observations revealed that WM tracts disruption were mainly concentrated in ipsilateral IFOF, SLF, and AF due to the tumor direct invasion in frontal gliomas patients with language impairment.^38^, ^39^, ^40^, ^41^ The current study further demonstrated microstructure abnormalities in contralateral IFOF, SLF, and AF, indicating that chronic damage induce by frontal DLGG may lead to fiber bundle degeneration at contralateral hemisphere.^42^

Interesting, the contralateral CST showed preserved integrity in GRE patients but damaged in non‐GRE patients. A potential explanation might be that the undamaged WM structure on contralateral CST may aid in the conduct of epileptic discharges. Further, when the subcortical structure containing the potential epicenter is damaged, the discharge tends to be isolated and less likely to form a generalized seizure. Besides, a previous study also demonstrated that the integrity of the ipsilateral CST is well preserved in glioma patients with motor epilepsy.^43^ Together with the current result, we assumed that the preserved microstructural integrity of the bilateral CST may be potential mechanisms underlying the generation and development of GRE.

### 4.2 Large‐scale network analysis

Using graph theory analysis, we identified significantly increased nodal efficiency predominantly located in the contralateral regions of the frontal–parietal (MFG, IPL and precuneus) and limbic networks (mOFC and cACC) in both patients group. Frontal–parietal network plays an important role in the control and management of executive functions, for example, the manipulation of information in working memory, reasoning, planning, problem‐solving and decision‐making.^44^, ^45^ As for limbic network, it involves in decision making and expectation. Based on literature, increased ALFF in contralateral hub of frontal–parietal network was reported in patients with frontal gliomas, indicating functional compensation within this network.^46^ Taken together, the higher nodal efficiency in contralateral frontal–parietal and limbic networks imply that highly functional plasticity might contribute to maintaining functional balance in GRE and non‐GRE patients.

However, lower nodal degree and betweenness in regions of the dorsal temporal lobe (superior or/and middle temporal gyrus) were observed in both patients group. This region is responsible for auditory and related multimodal sensory processing as well as auditory short‐term memory and language processing.^47^ The lower nodal degree and betweenness may be related to auditory and language processing impairments which were commonly observed for patients with left frontal glioma.^48^ Additionally, rMFG also showed lower nodal degree and betweenness in both patients group. rMFG is a key brain region for emotion regulation,^49^ altered nodal centrality in rMFG may lead to abnormal activation of the amygdala, resulting in excessive anxiety in patients with glioma.^50^

When compared GRE with non‐GRE, GRE patients showed further reductions in betweenness centrality in contralateral PCL, indicating that this node is susceptible to GRE. The PCL is mainly associated with motor and sensory innervations of the contralateral lower extremity. Prior neuroimaging work had demonstrated that paracentral regions emerging as potential epicenters of morphological abnormalities in idiopathic generalized epilepsy.^51^ Therefore, we speculate that decreased nodal betweenness in this node may contribute to epileptic seizures in GRE patients.

However, this study has certain limitations: firstly, a relatively small sample size from a single‐center was included in this study. In the future, larger samples from multi‐center will be needed to verify our results. In addition, the study was performed using only DTI analysis, which are insufficient to comprehend the brain WM plasticity induced GRE. Therefore, the integration of other technologies, such as magnetoencephalogram and fMRI, may provide valuable insights into functional and structural information for enriching our further understanding. Finally, because the language assessment was not available in our study, future work on acquiring language performance data and establishing the correlation with the altered WM parameters is warranted.

## 5 CONCLUSION

Using comprehensive set of sophisticated WM microstructural parameters, this study found that left frontal DLGG can induce complex WM reorganization concentrated in language, frontal–parietal and limbic networks. In addition, the preserved integrity in contralateral CST and server decreased betweenness centrality in PCL may be potential neuroimaging markers underlying the occurrence of presurgical seizures of GRE patients.

## AUTHOR CONTRIBUTIONS

Qiang Yue and Qiyong Gong conceived the project. Simin Zhang and Fei Zhao designed the protocol. Simin Zhang wrote the main manuscript. Simin Zhang, Shuang Li, Hanbin Shao, Xibiao Yang and Qiaoyue Tan obtained the data. Xibiao Yang reviewed MRI images of each patient. Simin Zhang and Fei Zhao analyzed the results. All authors critically reviewed the manuscript. Qiang Yue, and Qiyong Gong revised the manuscript.

## CONFLICT OF INTEREST STATEMENT

The authors declare no potential conflicts of interest.

## Supporting information


Data S1.

## Data Availability

The data that support the findings of this study are available on request from the corresponding author. The data are not publicly available due to privacy or ethical restrictions.
