# Supplementary material for: Multiparametric mapping of white matter reorganizations in patients with frontal glioma‐related epilepsy
Source: CNS Neurosci Ther. 2023 Jun 28;29(8):2366–76. doi: 10.1111/cns.14322 (PMC10352885; doi:10.1111/cns.14322)
Supplement: Supplementary file 1 — Data S1. [file CNS-29-2366-s001.docx]

**Multiparametric mapping of white matter reorganizations in patients with frontal glioma- related epilepsy**

**Supplementary Material**

## Supplementary Methods

### Participants


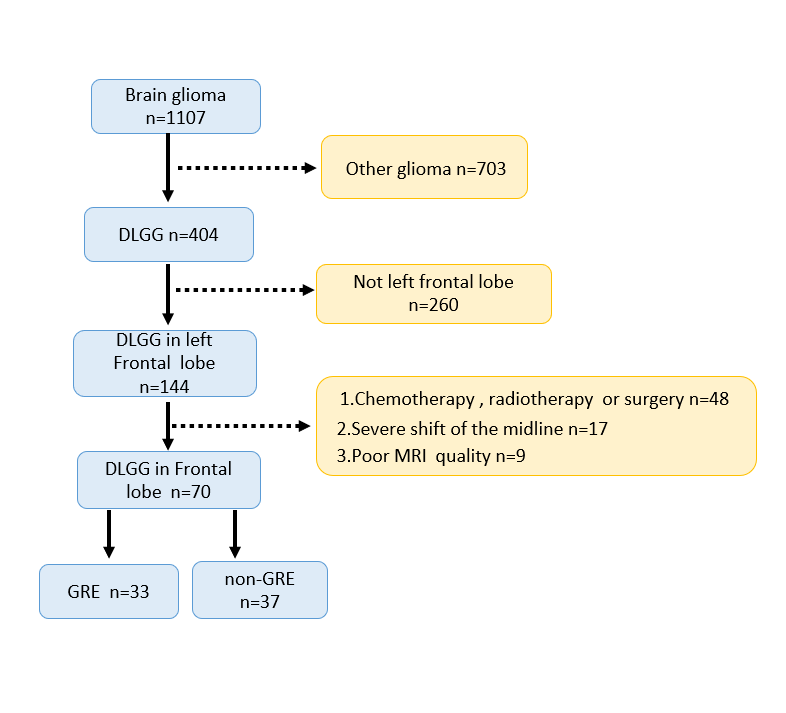


Fig. S1. Flow chart depicting patient’s enrollment process. DLGG= diffuse low-grade glioma; GRE= glioma-related epilepsy; non-GRE=patients without glioma-related epilepsy

### Glioma segmentation

The following were the steps involved in glioma segmentation: (1) the 3DT1WI volume was resampled to 1 mm3 isotropic resolution; (2) the brain was extracted using HD-BET [1] ( https://github.com/MIC-DKFZ/HD-BET) from the resampled 3DT1WI volume; (3) the remaining Gd-3DT1WI, FLAIR, and T2WI volumes were co-registered to the resampled 3DT1WI volume and multiplied with the brain mask generated by HD-BET; (4) using fslmerge, all four processed datasets were fused into four-dimensional images, which were then fed into a U-net trained on a brain tumor segmentation (BraTS) dataset [2], and a mask encompassing the entire glioma region was generated. Each mask was visually assessed independently by two neuroradiologists (Xibiao Yang and Hongjing Zhang, both with >10 years of experience in neuro oncology) using ITK-SNAP 3.6.0 software (http://www.itksnap.org). All the automated generated mask passed visual quality assurance.

### Tractseg

Along-tract FA statistics (Tractometry) were obtained as follows: Bundle-specific tractograms were generated using TractSeg [3, 4]. TractSeg employs machine learning to generate tract masks and tract orientation maps, enabling the accurate creation of bundle-specific tractograms. Subsequently, the FA was evaluated along each tract based on the method proposed by Chandio et al [5] : First, all streamlines were resampled to 100 segments and the FA was assessed at each segment of each streamline. Second, to determine each tract's centroid, QuickBundles was used. Now for each streamline each segment was assigned to the closest centroid segment. Finally, for each centroid segment the FA was averaged for all streamline segments that got assigned to this centroid segment resulting in 100 FA measurements per tract.

### Large-scale network analysis

After the brain tissue was extracted, a multi-tissue response function was estimated to compute the fiber orientation distributions utilizing the constrained spherical deconvolution (CSD) algorithm [6]. Five-tissue-type segmented T1 image and anatomically constrained tractography were utilized to generate 10 million whole-brain tractograms [7]. Tracts were truncated in this method if a poor structural termination was encountered, and the tracts were cropped at the grey matter white matter interface. To improve the biological plausibility of some streamline counts, spherical-deconvolution informed filtering of tractograms (SIFT) [8] was employed to filter the tracts to 5 million. These tracks were mapped into the 84 nodes to generate an 84 × 84 pair-wise connectivity matrix. Then, the GRETNA 2.0.0 (https://www.nitrc.org/projects/gretna/) was used to calculate the nodal topological metrics including nodal efficiency, degree centrality and betweenness centrality of the WM networks. Detailed definitions of topological properties are shown in Table S1.

Table S1. Summary of graph-theoretical metrics investigated in this study

| **Metrics** | **Features** | **Definitions** |
| --- | --- | --- |
| Nodal efficiency | Node efficiency defined as the inverse of the shortest path length between one node and other nodes measuring how efficient information is transferred between this node and the others. | 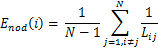  *E*_nod_ (*i*) measures the reciprocal of the average shortest path length between a given node *i* and all of the other nodes in the network. It quantifies the ability of node *i* in communication transfer within a network |
| Degree Centrality | Nodal degree is a common measure of centrality. For binary networks, the nodal degree is defined as the number of connections directly linked to a given node. For weighted networks, the nodal degree is defined as the sum of all the linking weights for a given node. | $d_{i}=\sum_{j\neq i\in G} w_{ij}$  where $w_{ij}$ is the edge weight linking between node *i* and node *j*, $d_{i}$ is the degree of node *i*. |
| Betweenness  Centrality | Betweenness centrality is defined as the fraction of all shortest paths in the network that pass through a given node, reflecting the nodes’ effects on information flow between other nodes. | 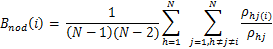  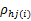 is the total numbers of the shortest path lengths between node *h* and *j* which passes through *h* for a specific node *i.* |

## Supplementary Results

Table S2. Regions exhibited altered topological properties along with their corresponding statistical values

| **Properties** | **Regions** | **ANCOVA** | |
| --- | --- | --- | --- |
|  |  | **Statistic (F value)** | ***p*-value** |
| Nodal efficiency | R. anterior caudal cingulate | 9.6695 | 0.00013981 |
|  | R. middle frontal | 10.3484 | 0.000079054 |
|  | R. medial orbitofrontal | 16.4204 | 0.000000624 |
|  | R. precuneus | 9.8234 | 0.00012279 |
|  | R. inferior parietal | 12.669 | 0.000011493 |
| Degree centrality | R. middle temporal | 11.8313 | 0.000023237 |
|  | R. paracentral | 19.3103 | 0.0000000722 |
|  | R. superior temporal | 21.177 | 0.0000000188 |
|  | R. rostral middle frontal | 9.6055 | 0.00014758 |
|  | R. superior parietal | 17.2543 | 0.00000033 |
| Betweenness centrality | R. middle temporal | 10.50554 | 0.0007 |
|  | R. paracentral | 21.97558 | 0.00000001 |
|  | R. rostral middle frontal | 17.79609 | 0.0000002 |

Note: using ANCOVA, setting age, gender and education level as covariates.

Table S3. Brain regions exhibited abnormal nodal efficiency among GRE, non- GRE and HC.

| Brain regions | Nodal efficiency | *P* value |
| --- | --- | --- |
| **GRE vs. HC** |  |  |
| R. anterior caudal cingulate | 0.120 ± 0.006 vs. 0.117 ± 0.004 | 0.025 |
| R. middle frontal | 0.128 ± 0.004 vs. 0.124 ± 0.003 | 0.00008 |
| R. medial orbitofrontal | 0.130 ± 0.003 vs. 0.129 ± 0.004 | 0.000004 |
| R. precuneus | 0.140 ± 0.004 vs. 0.136 ± 0.006 | 0.0003 |
| R. inferior parietal | 0.146 ± 0.004 vs. 0.141 ± 0.004 | 0.0007 |
| **non- GRE vs. HC** |  |  |
| R. anterior caudal cingulate | 0.122 ± 0.005 vs. 0.117 ± 0.004 | 0.0001 |
| R. middle frontal | 0.126 ± 0.004 vs. 0.124 ± 0.003 | 0.00004 |
| R. medial orbitofrontal | 0.135 ± 0.004 vs. 0.129 ± 0.004 | 0.00003 |
| R. precuneus | 0.139 ± 0.005 vs. 0.136 ± 0.006 | 0.0001 |
| R. inferior parietal | 0.145 ± 0.003 vs. 0.141 ± 0.004 | 0.00005 |
| **GRE vs. non- GRE** |  |  |
| NA | NA | NA |

Table S4. Brain regions exhibited abnormal degree centrality among GRE, non- GRE and HC.

| Brain regions | Degree centrality | *P* value |
| --- | --- | --- |
| **GRE vs. HC** |  |  |
| R. middle temporal | 1.803 ± 0.357 vs. 2.069 ± 0.177 | 0.00003 |
| R. paracentral | 1.863 ± 0.167 vs. 2.064 ± 0.135 | 0.000003 |
| R. superior temporal | 2.476 ± 0.400 vs. 2.738 ± 0.221 | 0.001 |
| R. rostral middle frontal | 2.963 ± 0.105 vs. 3.220 ± 0237 | 0.0007 |
| R. superior parietal | 3.007 ± 0.293 vs. 3.045 ± 0.273 | 0.00005 |
| **non- GRE vs. HC** |  |  |
| R. middle temporal | 1.852 ± 0.181 vs. 2.069 ± 0.177 | 0.0005 |
| R. paracentral | 1.846 ± 0.191 vs. 2.064 ± 0.135 | 0.00007 |
| R. superior temporal | 2.474 ± 0.284 vs. 2.738 ± 0.221 | 0.001 |
| R. rostral middle frontal | 2.763 ± 0.105 vs 3.220 ± 0237 | 0.00009 |
| R. superior parietal | 3.004 ± 0.273 vs. 3.045 ± 0.273 | 0.00002 |
| **GRE vs. non- GRE** |  |  |
| NA | NA | NA |

Tables S5. Brain regions exhibited abnormal betweenness centrality among GRE, non- GRE and HC.

| Brain regions | Betweenness centrality | *P* value |
| --- | --- | --- |
| **GRE vs. HC** |  |  |
| R. middle temporal | 3.321 ± 1.220 vs. 4.367 ± 1.142 | 0.0002 |
| R. rostral middle frontal | 4.243 ± 1.040 vs. 5.689 ± 1.245 | 0.000005 |
| R. paracentral | 9.670 ± 1.005 vs. 14.180 ± 2.733 | 0.000001 |
| **non- GRE vs. HC** |  |  |
| R. middle temporal | 3.350 ± 0.917 vs. 4.367 ± 1.142 | 0.0003 |
| R. rostral middle frontal | 3.937 ± 1.337 vs. 5.689 ± 1.245 | 0.00005 |
| R. paracentral | 12.670 ± 2.413 vs.14.180 ± 2.733 | 0.00007 |
| **GRE vs. non- GRE** |  |  |
| R. paracentral | 9.670 ± 1.005 vs.12.670 ± 2.413 | 0.00003 |

## Reference

1. Isensee F, Schell M, Pflueger I, et al. Automated brain extraction of multisequence MRI using artificial neural networks. Human Brain Mapping 2019; 40(17):4952-4964.

2. Isensee F, Kickingereder P, Wick W, Bendszus M, Maier-Hein KH: Brain Tumor Segmentation and Radiomics Survival Prediction: Contribution to the BRATS 2017 Challenge. In: *Brainlesion: Glioma, Multiple Sclerosis, Stroke and Traumatic Brain Injuries: 2018// 2018; Cham*: Springer International Publishing; 2018: 287-297.

3. Wasserthal J, Neher P, Maier-Hein KH. TractSeg - Fast and accurate white matter tract segmentation. Neuroimage 2018; 183:239-253.

4. Wasserthal J, Neher PF, Hirjak D, Maier-Hein KH. Combined tract segmentation and orientation mapping for bundle-specific tractography. Medical Image Analysis 2019; 58:101559.

5. Chandio B, Harezlak J, Koudoro S, Reagan D, Garyfallidis E: Bundle Analytics: a computational and statistical analyses framework for tractometric studies; 2019. Paper presented at the Organization for Human Brain Mapping.

6. Tournier JD, Calamante F, Connelly A. Robust determination of the fibre orientation distribution in diffusion MRI: Non-negativity constrained super-resolved spherical deconvolution. Neuroimage 2007; 35(4):1459-1472.

7. Smith RE, Tournier JD, Calamante F, Connelly A. Anatomically-constrained tractography: improved diffusion MRI streamlines tractography through effective use of anatomical information. Neuroimage 2012; 62(3):1924-1938.

8. Smith RE, Tournier JD, Calamante F, Connelly A. SIFT: Spherical-deconvolution informed filtering of tractograms. Neuroimage 2013; 67:298-312.
